# Medical, Administrative, Financial, and Social Challenges in the Management of a Case of Hemolytic Disease of the Fetus Caused by a Rare Alloantibody in a Low-Resource Setting

**DOI:** 10.7759/cureus.59676

**Published:** 2024-05-05

**Authors:** K Aparna Sharma, Hem Pandey, Nilanchali Singh, Deepali Garg, Shainy P, Vatsla Dadhwal, Anubhuti Rana, Priyanka Chaudhary

**Affiliations:** 1 Obstetrics and Gynecology, All India Institute of Medical Sciences, New Delhi, New Delhi, IND; 2 Transfusion Medicine, All India Institute of Medical Sciences, New Delhi, New Delhi, IND

**Keywords:** rh isoimmunized, intrauterine transfusion, atypical antibodies, rh 17, hemolytic disease of fetus

## Abstract

Antibodies to high-frequency antigens are rarely implicated in cases of hemolytic disease of the fetus and newborn (HDFN), yet they pose a challenge to both clinical staff and transfusion medicine, especially with the identification of the implicating antibody and the arrangement of compatible blood for intrauterine transfusion. Here we report one such interesting case of HDFN caused by an alloantibody to a high-frequency antigen belonging to the Rhesus (Rh) blood group system. The patient presented at the 19th week with Rh-isoimmunized pregnancy. She received six intrauterine transfusions (IUTs) at different intervals during the antenatal period. Arranging the blood of this rare blood group required great efforts from hospital administration, clinicians, and social workers. At 31 weeks, the fetus developed a non-reassuring non-stress test (NST). Hence, the baby was delivered by cesarean section. The baby fared well in the neonatal period. With great efforts and support from social health workers, the Japanese Red Cross society, the administration, and non-government organizations, the impossible became possible.

## Introduction

Hemolytic disease of the fetus and newborn is an important cause of fetal morbidity and mortality in the antenatal period and immediate postnatal period [1]. Antibodies to the Rhesus (Rh) blood group system have been implicated in the majority of cases [2]. Unlike developed countries, antibodies to the RH1 (anti-D) blood group are still implicated in the majority of cases of HDFN in our country, despite the presence of preventive Rh immunoglobulin [3]. This is followed by antibodies against other Rh antigens, notably RH2, RH3, RH4, and RH5, with a small fraction of these cases caused by antibodies to other minor blood group antigens [4-6]. The availability of intrauterine transfusions (IUT) has contributed to the successful outcomes in the majority of these cases [7].

Hemolytic disease of the fetus and newborn (HDFN) rarely implicates antibodies to high-frequency antigens, yet they pose a challenge to both clinical staff and transfusion medicine, particularly in identifying the implicating antibody and arranging compatible blood for intrauterine transfusion. Here, we present an intriguing case of HDFN resulting from an alloantibody to a high-frequency antigen in the RH blood group system, highlighting several crucial issues in managing such cases.

## Case presentation

The patient first presented to our center in 2018 as a referred case from another center with a history of severe anemia (Hb 6.5 g/dl) and fetal intrauterine death (day seven). The patient had no living issue. Out of her prior five pregnancies, four of them had intrauterine deaths at 28-36 weeks of gestation, and one was a spontaneous abortion at 10 weeks. She had fetal growth restriction and eclampsia in her first pregnancy, leading to stillbirth. Thereafter, fetal hydrops were noted in others. All the pregnancies were managed at primary or secondary-level centers. She had never received a blood transfusion before.

The referring center reported that they were unable to find a compatible unit for the patient despite cross-matching numerous units. Due to the unavailability of compatible units, it was decided to reduce the antibody titer by serial plasma exchanges followed by transfusion of incompatible units under high-dose intravenous immunoglobulin (IVIG) if required. Two serial plasma exchanges (1.5 volumes each) on alternate days reduced the titer to 1:64 from 1:124. She delivered a macerated male hydropic baby of 748 grams vaginally but didn’t require any blood transfusion. She was advised to take an iron-rich diet and to visit for preconception counseling with the department of obstetrics as well as transfusion medicine before any further pregnancy. The option of autologous transfusion (autologous frozen red cells) for the management of maternal needs in subsequent pregnancies was considered. She, however, did not report her seventh pregnancy and had an abortion at 10 weeks.

In her eighth and present pregnancy, the patient reported at the 19th week for antenatal booking. Ultrasound was done to evaluate fetal anatomy, and fetal anemia was evaluated by Doppler ultrasonography of the middle cerebral artery- peak systolic velocity (MCA-PSV). The fetus was found to have an MCA-PSV of more than 1.5 MoM with no evidence of hydrops. The patient was admitted to our center, where she initially received two cycles of IVIG (0.5 g/kg body weight; a total of 25g), one week apart. No complications related to IVIG administration were noted. She was started on aspirin 150 mg/day for preeclampsia prevention.

Investigations

During the immuno-hematological workup, the blood group of the patient was found to be B RhD positive, and pan-reactivity (uniform, 4+) was observed with both 3-cell and 11-cell panels with a negative auto-control. Phenotyping for minor blood group antigens revealed the absence of RH2, RH3, RH4, and RH5 antigens, and thus a presumptive diagnosis of the D-- phenotype with an anti-Rh17 antibody was made. The patient's sample was referred to the International Blood Group Reference Laboratory, Bristol, UK, which confirmed the patient's Rh phenotype as D- C- c- E- e- Rh17- Rh 29+. Genomic DNA sequencing revealed the presence of the hybrid allele RHCE*CE(1-2)D(3-8)CE(9-10). The titer of the antibody with R1R1 cells was found to be 1024.

Differential diagnosis

The complete workup for earlier fetuses was done for hydrops fetalis, and it was already known in this pregnancy that this was a hydropic fetus due to Rh-isoimmunized pregnancy.

Treatment

As there is no rare donor registry in India, the International Rare Donor Panel was contacted to arrange O D-- phenotype red cell units for intrauterine transfusion. A blood donor from India was registered on the panel, but he refused to donate blood. As frequent intrauterine transfusions would have been required at different intervals, we contacted the Japanese Red Cross, which has registered multiple donors of the O D-- phenotype. Four units of the O D-- phenotype red cell units were transported from the Japanese Red Cross to our center at different time periods during the pregnancy for intrauterine transfusion. We could also identify one blood donor in Lucknow, India, of B D-- phenotype, and the blood was transported to our center in Delhi.

The first IUT was done at 21+6 weeks of gestation, with leucocyte-reduced red cell units of O Rh D-- phenotype received from the Japanese Red Cross. The unit was irradiated and processed to a hematocrit of 75%. The blood group of the fetus was found to be B Rh D positive in the sample collected before IUT. Subsequently, the mother received five IUTs at different intervals during the antenatal period. The last IUT was given at 28+1 weeks of gestation. Maternal anemia was treated with oral hematinic therapy. Steroid cover for fetal lung maturity was given at 28 weeks of gestation. Maternal and fetal surveillance was continued. At 31 weeks, the fetus developed a non-reassuring non-stress test (NST). Despite resuscitative measures, the fetus continued to show persistent, non-reassuring NST. The cardiotocography showed a recurrent sinusoidal pattern with a shift of baseline from 150 bpm to 120 bpm with absent acceleration. The biophysical profile showed an absent fetal tone and breathing movements. Hence, it was planned to deliver the baby at 31 weeks of gestation by cesarean section.

Outcome and follow-up

As only two units of O RhD-- phenotype red cells were available with us, which may have been required for exchange transfusion of the baby post-delivery, one unit of autologous blood for maternal need was collected using the pre-operative hemodilution technique. The C-section was uneventful, and a healthy female baby weighing 1415 grams was delivered. The blood was re-transfused post-lower segment cesarian section (LSCS). Appearance, Pulse, Grimace, Activity, and Respiration (APGAR) scores at birth and five minutes were 8 and 9, respectively. Her cord bilirubin was 5.9 mg/dl, and her hematocrit was 20.1%. The baby was shifted to the neonatal ICU, where she received early surfactant rescue therapy, one cycle of partial exchange transfusion, and six cycles of double surface phototherapy. The baby was also treated with laser therapy for retinopathy of prematurity. The total duration of the baby's stay in the neonatal ICU was 29 days. The baby fared well in the neonatal period and was discharged in satisfactory condition.

Support from the social service department and administration

To transport blood from various national and international facilities, monetary support was required. The medical social officers are indispensable members of the ‘Multi-professional Health Care Team’ at our institute. They worked relentlessly to arrange for funds. Many government schemes are available to help patients with below-the-poverty line status. However, the procedure was complex, as either the rules were usually for patients and not for the unborn fetuses, or overseas transportation was not a part of the policy. Therefore, for the first installment, they arranged for help from a non-government organization (NGO). For the second installment, too, an NGO supported the funds. The third and fourth installments were supported by the government scheme, Rashtriya Arogya Nidhi (RAN). Under RAN, the funds are released as a ‘one-time grant’. Financial assistance for treatment up to 2735 USD is provided. Powers are delegated to an institute's medical superintendent or director for treatment up to 6840 USD.

After arranging for funds, exhaustive documentation was required, comprising the original import permit (Drug Controller Gen. of India), original custom duty exemption certificate, bank authorized dealer code, patient self-attested copy of identification, and a personal name authorization letter to the courier company with legal formalities. The administration efficiently cleared the necessary administrative formalities, and a blood bag from Japan was brought to the transfusion laboratory.

## Discussion

The prevalence of D-- phenotype has not been reported from India, and only one case of D-- pregnancy has been reported from the country to date [8]. Intervention in the antenatal period was not required in the case reported previously, and HDFN was identified only after delivery when fetal distress and hyperbilirubinemia were identified in the baby. In the present case, we could identify the blood group of the patient and the presence of anti-Rh17 even before the patient conceived the baby. The severity and hemolytic potential of the antibody was also known, considering that the patient had lost previous fetuses mostly during the second trimester. Globally, 18 cases of Rh-- pregnancy with anti-Rh17 have been reported, out of which eight pregnancies were reported with successful outcomes.

The primary pillars of management of pregnancies complicated by rare antibodies such as anti-Rh17 have been plasma exchanges [4,5,9], and intravenous immunoglobulins primarily until the 20th week of gestation, and subsequently intrauterine transfusions in the later part of the pregnancy. The former is assumed to reduce the amount of alloantibodies in the maternal plasma and limit the extent of fetal hemolysis until intrauterine transfusions are technically feasible. Acute hemodynamic changes that occur after intrauterine transfusions are known to be less tolerated by fetuses below the 20-week period of gestation (POG) [4]. Zweirs C et al. from the Netherlands (2017) reported procedure-related fetal demise in < 20 weeks POG as 8.5% compared to 0.9% in fetuses less than 20 weeks POG (P = 0.001) [10].

The patient presented to our center at the 19th week of gestation, and thus plasma exchange was not considered an option in our case. As the fetus was already anemic at presentation, it was decided to start intravenous immunoglobulins until compatible red cell units could be arranged. Arranging D-- red cell units presented a challenge due to the lack of a national rare donor registry or a known blood donor of the rare phenotype. The rare phenotype units could be arranged on time with the support of the International Rare Donor Panel and the Japanese Red Cross. The present case thus highlights the significance of rare donor panels and the need for the development of rare donor registries at the national level in our country, which would have helped in the timely arrangement of compatible units.

As the blood units were not available in the country and needed to be arranged from Japan, it became challenging to decide the right time for the arrangement of the blood. This was due to the fact that multiple IUTs are required at different intervals in the antenatal period, and a minimum duration of 10-14 days was required for the collection, testing, and transportation of blood. An early arrangement would lead to the red cells getting older, making them comparatively unsuitable for IUTs, whereas a late arrangement carried a chance of fetal harm. This was also complicated by the international travel restrictions implemented at the time due to the COVID-19 pandemic, which restricted the number of flights available for the transportation of blood. Due to these difficulties, we utilized the remaining aliquots of already-arranged blood units, which were much older than recommended, especially in the early neonatal period, where frequent but smaller volumes of blood needed to be transfused. The supernatant from the older units was removed by centrifugation completely, and no immediate adverse reactions were noted in the fetus post-transfusion in the antenatal period. Another aspect of the present case that we would like to highlight is the financial support that was required for arranging the units from Japan. The social service department of the institute played a vital role in this aspect.

## Conclusions

This case is unique as it is not only the first successfully managed case of RhD-isoimmunized pregnancy in the country but also a unique case where the social support team, clinicians, administration, and international support played a key role in providing a favorable outcome. Establishing a rare blood group registry in developing nations is the need of the hour, as procuring rare blood was the biggest hurdle.
